# Serum Zinc Concentration Correlates With Ferritin Concentration in Patients Undergoing Peritoneal Dialysis: A Cross-Sectional Study

**DOI:** 10.3389/fmed.2020.537586

**Published:** 2020-09-17

**Authors:** Shohei Kaneko, Junki Morino, Saori Minato, Katsunori Yanai, Yuko Mutsuyoshi, Hiroki Ishii, Momoko Matsuyama, Taisuke Kitano, Mitsutoshi Shindo, Akinori Aomatsu, Haruhisa Miyazawa, Yuichiro Ueda, Kiyonori Ito, Keiji Hirai, Susumu Ookawara, Yoshiyuki Morishita

**Affiliations:** Division of Nephrology, First Department of Integrated Medicine, Saitama Medical Center, Jichi Medical University, Saitama, Japan

**Keywords:** zinc, ferritin, peritoneal dialysis, dietary restriction, malabsorption

## Abstract

**Background:** Zinc deficiency is common and is associated with erythropoietin resistant anemia, dysgeusia, and hypogonadism in patients undergoing hemodialysis. However, the prevalence and clinical effects of zinc deficiency in patients undergoing peritoneal dialysis (PD) have not been determined.

**Methods:** This was a retrospective, cross-sectional study. The prevalence of serum zinc deficiency and the clinical factors related to serum zinc concentration were determined in 49 patients undergoing PD [mean age 59.5 years (±14.8 years), 38/49 were men (78.6%), median PD period 24.0 months (12.5–45.0 months)]. A serum zinc concentration <60 μg/dL was defined as serum zinc deficiency, and a serum zinc concentration between 60 and 80 μg/dL as possible serum zinc deficiency.

**Results:** Serum zinc deficiency was present in 51% (25/49) of the patients, and possible serum zinc deficiency was present in 45% (22/49) of patients undergoing PD. Multivariate analysis showed that serum zinc concentration significantly correlated with serum ferritin concentration (β = 0.357, *P* < 0.01).

**Conclusions:** The prevalences of serum zinc deficiency and possible serum deficiency are high and serum zinc concentration correlates with serum ferritin concentration in patients undergoing PD.

## Introduction

Zinc is an essential trace element and is required for important physiological functions, such as immunity, nerve function, growth, skeletal integrity, and endocrine function (1–6). Clinically, zinc deficiency induces various pathological conditions, such as dermatitis, anemia, susceptibility to infection, gastrointestinal disorders, including loss of appetite, dysgeusia, and diarrhea, developmental disorders, and hypogonadism (1–6).

A number of studies have reported a high prevalence of zinc deficiency, and zinc supplementation ameliorates several clinical problems related to zinc deficiency, such as erythropoietin resistant anemia, dysgeusia, and hypogonadism, in patients undergoing hemodialysis (7–15). The causes of zinc deficiency in patients undergoing hemodialysis are thought to be multiple, and include dietary restriction attributable to renal failure, malabsorption, loss into the dialysate, and the effects of oral medications, such as phosphate binders (3, 7, 12, 16–19). However, there have been few studies of zinc deficiency in patients undergoing peritoneal dialysis (PD) (20, 21). In addition, the clinical factors that are associated with zinc status in patients undergoing PD have not been investigated. Therefore, in the present study, we determined the prevalence of zinc deficiency and the clinical factors associated with serum zinc concentration in patients undergoing PD.

## Materials and Methods

### Study Design

This was a retrospective cross-sectional study of the prevalence of zinc deficiency and the clinical factors associated with zinc status in patients undergoing PD.

### Study Population

This study was conducted at Saitama Medical Center, Jichi Medical University. We studied patients who were undergoing PD between April 2018 and March 2019. The inclusion criteria were that the patients were undergoing PD, that they were >18 years old, and they had their serum zinc concentration measured for clinical purposes among all patients undergoing PD in our hospital. The exclusion criteria were zinc supplementation, renal transplantation, and lack of consent to participate in the study. All of the patients had been treated according to the 2009 Japanese Society for Dialysis Therapy Guidelines for PD and the 2015 Japanese Society for Dialysis Therapy Guidelines for Renal Anemia in Chronic Kidney Disease (22, 23). Because this study was observational and retrospective, an opt-out procedure replaced the need for the provision of written consent. This study was approved by the Saitama Medical Center, Jichi Medical University Ethics committee (DAI-RIN 15-34) and was conducted in accordance with the principles of the Declaration of Helsinki.

### Data Collection

Data from patient medical records were used for this analysis. Information regarding the age, sex, body mass index, PD period, combination hemodialysis therapy, oral medication (antacids, phosphate binders, vitamin D and loop diuretics), intravenous iron supplementation, oral iron supplementation, PD modality [continuous ambulatory peritoneal dialysis (CAPD) and automated peritoneal dialysis (APD)], total weekly Kt/V urea, peritoneal weekly Kt/V urea, renal weekly Kt/V urea, 4-h dialysate/plasma creatinine ratio, and laboratory measurements, including serum zinc concentration was obtained for each patient. Information regarding the symptoms associated with zinc deficiency (dysgeusia, dermatitis, and chronic diarrhea) was obtained from medical records. Combination hemodialysis therapy was defined as a combination of hemodialysis once a week in addition to PD. Oral iron supplementation was defined as the administration of ferrous citrate or an iron-containing phosphate binder. CAPD is a method of changing PD fluids manually and APD is a method of changing PD fluids using an automated system (24–27). Peritoneal weekly Kt/V urea is an indicator of the efficacy of PD and renal weekly Kt/V is an indicator of residual renal function (28); they were summarized together as total weekly Kt/V urea (28). Four-hour dialysate/plasma creatinine ratio is used as an indicator of peritoneal permeability (29). The Geriatric Nutritional Risk Index (GNRI) is an indicator of nutritional status calculated by (14.89 × serum albumin [g/dL]) + 41.7 × (body weight/ideal body weight [kg]) (30). Blood parameters and PD fluid parameters were measured by the Department of Clinical Laboratory, Saitama Medical Center.

### Definition of Zinc Deficiency

On the basis of the Japanese guidelines for zinc deficiency, a serum zinc concentration <60 μg/dL was defined as serum zinc deficiency, a serum zinc concentration between 60 and 80 μg/dL as possible serum zinc deficiency, and >80 μg/dL as normal (31). These guidelines were published by the Japanese Society of Clinical Nutrition in 2018 (31).

### Definition of Erythropoietin Resistance Index

The resistance of erythropoietin stimulating agents to anemia in patients undergoing dialysis was evaluated as erythropoietin resistance index (32, 33). The erythropoietin resistance index was defined as the average weekly dose of recombinant human erythropoietin (IU)/body weight (kg)/hemoglobin (g/dL) (32, 33). The ratio of recombinant human erythropoietin darbepoetin alfa or epoetin beta pegol was converted to 200:1 (34).

### Statistical Analysis

The normality of distribution of the data was assessed using the Shapiro–Wilk test. Quantitative variables are expressed as mean ± standard deviation (SD) for normally distributed data and as median and interquartile range [IQR] for non-normally distributed data. Categorical variables are expressed as frequency and percentage. Univariate and multivariate regression analyses were performed to determine the clinical factors associated with serum zinc concentration. Statistical analysis was performed using JMP (SAS Institute Inc., Cary, NC, USA). *P* < 0.05 was considered to represent statistical significance.

## Results

### Clinical Characteristics of the Patients

Forty-nine participants were enrolled in the present study. Table 1 shows the clinical characteristics of the study population. The mean age of the participants was 59.5 ± 14.8 years old. Thirty-eight (78.6%) men were enrolled in the study. The participants' median body mass index was 23.6 [20.8–25.4] kg/m^2^, their median PD period was 23.6 [20.8–25.4] months, and their mean serum zinc concentration was 60.1 ± 10.4 μg/dL. None of the participants had received intravenous iron supplementation, but 27 (55.1%) had received oral iron supplementation. With respect to the PD modality, 18 (36.7%) participants had undergone CAPD and 31 (63.3%) had undergone APD. Eight percent (4/49) of the participants had dysgeusia, 18% (9/49) had dermatitis, and 4% (2/49) had chronic diarrhea. The mean GNRI was 95.3 ± 10.8 (g/dL). The mean erythropoietin resistance index was 6.2 ± 3.7 [IU/week/kg/(g/dL)]. Table 2 and shows the prevalence of serum zinc deficiency and possible serum zinc deficiency in participants who had undergone PD. In this sample, 51% (25/49) of the participants had serum zinc deficiency (serum zinc concentration < 60 μg/dL), 45% (22/49) had possible serum zinc deficiency (60 ≤ serum zinc concentration < 80 μg/dL), and 4% (2/49) had normal zinc concentrations (serum zinc concentration ≥ 80 μg/dL).

**Table 1:** Characteristics of the study population (*n* = 49).

**Variables**	**Mean ± SD or median [IQR]**	**Number (%)**
Age (years)	59.5 ± 14.8	
Sex male		38 (78.6)
Body mass index (kg/m^2^)	23.6 [20.8–25.4]	
PD period (months)	24 [13–45]	
Hemodialysis combination		12 (24.5)
Antacids		19 (38.8)
Phosphate binder		44 (89.8)
Vitamin D		27 (55.1)
Diuretics		25 (51.0)
Intravenous iron supplementation		0 (0)
Oral iron supplementation		27 (55.1)
Dysgeusia		4 (8.2)
Dermatitis		9 (18.4)
Chronic diarrhea		2 (4.1)
PD modality: CAPD		18 (36.7)
PD modality: APD		31 (63.3)
Red blood cells (10^4^/μL)	350.8 ± 46.9	
White blood cells (10^3^/μL)	6.3 [5.2–7.5]	
Hemoglobin (g/dL)	10.9 ± 1.4	
Platelets ( ×10^4^/μL)	23.0 ± 7.5	
Total protein (g/dL)	6.2 ± 0.5	
Albumin (g/dL)	3.4 ± 0.5	
Blood urea nitrogen (mg/dL)	53.0 [48.5–63.5]	
Creatinine (mg/dL)	11.1 ± 3.2	
C-reactive protein (mg/dL)	0.11 [0.04–0.33]	
Uric acid (mg/dL)	6.0 [5.1–7.2]	
Sodium (mEq/L)	138.1 ± 3.8	
Potassium (mEq/L)	4.6 ± 0.1	
Chloride (mEq/L)	100.2 ± 5.0	
Corrected calcium (mg/dL)	9.1 ± 0.6	
Phosphorus (mg/dL)	5.6 ± 1.1	
Zinc (μg/dL)	60.1 ± 10.4	
Magnesium (mg/dL)	2.1 [1.9–2.5]	
Copper (μg/dL)	91 [81–102]	
Iron (μg/dL)	75 [59–97]	
Transferrin saturation (%)	31.5 [22.8–38.8]	
Ferritin (mg/mL)	125.1 [79.9–233.1]	
Blood glucose (mg/dL)	109 [98–132]	
HbA1c (%)	5.5 [4.9–6.1]	
Total cholesterol (mg/dL)	170.9 ± 45.2	
HDL cholesterol (mg/dL)	46.7 ± 14.2	
LDL cholesterol (mg/dL)	91.8 ± 36.0	
Triglycerides (mg/dL)	113.0 [81.0–165.5]	
β_2_ microgloblin (mg/L)	28.4 ± 9.2	
Intact PTH (pg/dL)	236.0 ± 133.2	
Total Kt/V urea	1.6 [1.3–1.7]	
Peritoneal Kt/V urea	1.1 ± 0.5	
Renal Kt/V urea	0.5 ± 0.3	
D/P Creatinine	0.65 ± 0.14	
Dose of ESA (IU)	20,000 [10,000–30,000]	
ERI [IU/week/kg/(g/dL)]	6.2 ± 3.7	
GNRI (g/dL)	95.3 ± 10.8	

*SD, standard deviation; IQR, interquartile range; PD, peritoneal dialysis; CAPD, continuous ambulatory peritoneal dialysis; APD, automated peritoneal dialysis; HbA1c, hemoglobin A1c; HDL, high-density lipoprotein; LDL, low-density lipoprotein; PTH, parathormone; D/P Cre, 4-h dialysate/plasma creatinine ratio; ESA, erythropoietin stimulating agents; ERI, erythropoietin resistance index; GNRI, geriatric nutritional risk index*.

**Table 2:** Prevalence of zinc deficiency in patients undergoing PD (*n* = 49).

**Zinc status**	**Number (%)**
Serum zinc deficiency (serum zinc level < 60 μg/dL)	25 (51)
Potential serum zinc deficiency (60 ≤ serum zinc level < 80 μg/dL)	22 (45)
Normal (80 μg/dL ≤ zinc level)	2 (4)

*PD, peritoneal dialysis*.

### Factors Associated With Serum Zinc Concentration in Patients Undergoing PD

Table 3 shows the correlations between serum zinc concentration and other clinical parameters in patients undergoing PD. In univariate analysis, serum total protein (β = 0.357, *P* = 0.01), albumin (β = 0.314, *P* = 0.03), triglyceride (β = 0.300, *P* = 0.04), and ferritin (β = 0.435, *P* < 0.01) concentrations significantly correlated with zinc concentration. The correlation between erythropoietin resistance index and serum zinc concentration was not observed. No significant correlations between the symptoms related to zinc deficiency (dysgeusia, dermatitis, and chronic diarrhea) and serum zinc concentration were observed. In multiple regression analysis using these factors as explanatory variables, ferritin concentration (β = 0.357, *P* < 0.01) significantly correlated with zinc concentration (Table 3), but there were no correlations with other parameters.

**Table 3:** Factors associated with serum zinc concentration in patients undergoing PD (*n* = 49).

**Variables**	**Univariate**		**Multivariate**	
	**β**	***P*-value**	**β**	***P*-value**
Age	−0.184	0.20		
Sex male	−0.123	0.40		
Body mass index	−0.106	0.47		
PD period	0.076	0.60		
Hemodialysis combination	−0.070	0.63		
Antacids	0.017	0.91		
Phosphate binder	−0.240	0.10		
Vitamin D	0.033	0.82		
Diuretics	−0.038	0.80		
Oral iron supplementation	−0.023	0.88		
Dysgeusia	0.106	0.47		
Dermatitis	−0.102	0.48		
Chronic diarrhea	0.088	0.55		
PD modality: CAPD	0.046	0.75		
PD modality: APD	−0.046	0.75		
Red blood cells	−0.151	0.30		
White blood cells	0.083	0.57		
Hemoglobin	−0.120	0.41		
Platelets	0.120	0.41		
Total protein	0.357	0.01	0.105	0.57
Albumin	0.314	0.03	0.208	0.23
Blood urea nitrogen	−0.117	0.42		
Creatinine	0.166	0.25		
C-reactive protein	0.129	0.38		
Uric acid	0.060	0.68		
Sodium	−0.009	0.95		
Potassium	0.055	0.71		
Chloride	−0.135	0.36		
Corrected calcium	0.007	0.96		
Phosphorus	−0.143	0.33		
Magnesium	−0.122	0.40		
Copper	0.040	0.79		
Iron	−0.027	0.85		
Transferrin saturation	−0.068	0.64		
Ferritin	0.435	<0.01	0.357	<0.01
Blood glucose	−0.049	0.74		
HbA1c	−0.089	0.54		
Total cholesterol	0.269	0.06		
HDL cholesterol	−0.044	0.76		
LDL cholesterol	0.159	0.28		
Triglycerides	0.300	0.04	0.162	0.26
β2 microgloblin	0.056	0.70		
Intact PTH	0.060	0.68		
Total Kt/V urea	−0.226	0.12		
Peritoneal Kt/V urea	−0.021	0.89		
Renal Kt/V urea	0.169	0.25		
D/P Creatinine	−0.061	0.67		
Dose of ESA	0.070	0.64		
ERI	−0.186	0.21		
GNRI	0.131	0.37		

*SD, standard deviation; IQR, interquartile range; PD, peritoneal dialysis; CAPD, continuous ambulatory peritoneal dialysis; APD, automated peritoneal dialysis; HbA1c, hemoglobin A1c; HDL, high-density lipoprotein; LDL, low-density lipoprotein; PTH, parathormone; D/P Cre, 4-h dialysate/plasma creatinine ratio; ESA, erythropoietin stimulating agents; ERI, erythropoietin resistance index; GNRI, geriatric nutritional risk index*.

## Discussion

This study shows that serum zinc deficiency (51%) and possible serum zinc deficiency (45%) are highly prevalent in patients undergoing PD, which consistent with the results of previous studies (20, 21). In addition, we found a significant correlation between serum zinc and ferritin concentrations after excluding confounders in patients undergoing PD. This is the first study to show a positive correlation between zinc and ferritin status in patients undergoing PD.

The prevalences of serum zinc deficiency and possible serum zinc deficiency in patients undergoing PD were high in the present study. Several factors, such as dietary restriction attributable to renal failure (3, 7, 21), lower intestinal zinc absorption (18, 19), and the use of phosphate binders, which are known to inhibit the absorption of zinc in the gastrointestinal tract (16, 17, 21), may contribute to high prevalence of zinc deficiency in patients undergoing PD. In addition, there was a significant correlation between serum zinc and ferritin concentrations after excluding confounders in multivariate analysis. This may be explained by several mechanisms. dietary restriction and malabsorption due to renal failure may affect cause deficiencies of both zinc and iron, but greater zinc usage may also contribute to this positive correlation. Zinc has been reported to be used for the biosynthesis of protoporphyrin IX, a precursor of hemoglobin, instead of iron during iron deficiency (35). Furthermore, there have been several reports of a positive association between serum zinc and ferritin concentrations in adult and infant patients who were not undergoing dialysis (36, 37); however, the mechanism of this association has not been fully determined. Therefore, further studies are required to identify the mechanism of the zinc-ferritin correlation identified in patients undergoing PD. In clinical setting, regular measurement of serum ferritin concentration is recommended for the treatment in patients undergoing dialysis (38). However, serum zinc concentration is only measured when the patient has characteristic symptoms. Our study suggests that iron and zinc are positively correlated in patients undergoing peritoneal dialysis. Therefore, clinicians should be note of the potential presence of patients with zinc deficiency among the patients with iron deficiency.

In this study, 8% (4/49) of the participants had dysgeusia, 18% (9/49) had dermatitis, and 4% (2/49) had chronic diarrhea; however, significant correlations were not observed between these symptoms and serum zinc concentration. This may be because these symptoms in patients undergoing PD were caused by various abnormalities such as uremia, volume overload, electrolyte abnormalities, and prescribed drugs. Further prospective and interventional studies are needed to clarify the relationship between serum zinc concentration and symptoms in these populations. Additionally, the serum zinc concentration was not associated with total protein levels, albumin levels, the GNRI, and C-reactive protein levels on multivariate analysis in this study. These results suggested that the serum zinc level was not associated with malnutrition and inflammatory syndrome in patients undergoing PD. Future large-scale studies are necessary to investigate the relationships among zinc deficiency, malnutrition, and inflammatory syndrome in patients undergoing PD.

The Japanese Society of Clinical Nutrition guidelines recommend oral zinc supplementation for patients with characteristic symptoms and low zinc status (serum zinc deficiency or possible serum zinc deficiency) (31). In addition, these guidelines emphasize the importance of monitoring the improvement of symptoms during zinc supplementation (31). Current guidelines recommend oral zinc supplementation at a dose of 50–100 mg/day for adult patients with zinc deficiency (31). They also recommend oral zinc supplementation at a dose of 1–3 mg/kg/day (otherwise, 25 mg/day if the body weight is <20 kg or 50 mg/day if the body weight is more than 20 kg) for infant patients (31). Thus, further interventional studies are needed to verify the benefits of zinc supplementation in patients undergoing PD.

There were several limitations in this study. First, it was performed at a single facility and it is unclear whether the results are generalizable to other populations. Second, the sample size of this study was small. Third, because this was a cross-sectional study, this study did not show an association between clinical outcomes, such as morbidity and mortality, and zinc deficiency. Future long-term observational studies are necessary to investigate zinc deficiency and outcomes in patients undergoing PD. Thus, further multicenter-interventional studies are required to identify zinc deficiency and determine the effects of zinc supplementation in patients undergoing PD.

## Conclusion

The prevalences of serum zinc deficiency and possible serum zinc deficiency are high and serum zinc concentration significantly positively correlates with ferritin concentration in patients undergoing PD. Clinicians should be aware of this when they manage such patients.

## Data Availability Statement

The datasets analyzed in this article are not publicly available. Requests to access the datasets should be directed to Yoshiyuki Morishita, ymori@jichi.ac.jp.

## Ethics Statement

The studies involving human participants were reviewed and approved by Saitama Medical Center, Jichi Medical University Ethics committee (DAI-RIN 15-34). Written informed consent for participation was not required for this study in accordance with the national legislation and the institutional requirements.

## Author Contributions

SK analyzed data and wrote the manuscript. SK, JM, SM, KY, YMu, HI, MM, TK, MS, AA, HM, YU, KI, KH, SO, and YMo contributed to the study design, data collection, and statistical analyses. YMo supervised this study. All authors read and approved the final version of the manuscript.

## Conflict of Interest

The authors declare that the research was conducted in the absence of any commercial or financial relationships that could be construed as a potential conflict of interest.

## Abbreviations

APD: Automated peritoneal dialysis
CAPD: Continuous ambulatory peritoneal dialysis
GNRI: Geriatric nutritional risk index
IQR: Interquartile range
PD: Peritoneal dialysis
SD: Standard deviation.
